# Continuous Glucose Monitoring Data Compression Using Peak-Nadir Encoding in Diabetes: Method Development and Evaluation

**DOI:** 10.2196/91959

**Published:** 2026-07-20

**Authors:** Clara Bender, Line Davidsen, Søren Schou Olesen, Simon Cichosz

**Affiliations:** 1 Department of Health Science and Technology, Aalborg University, Selma Lagerløfs Vej 249, Gistrup, North Denmark, 9260, Denmark, 45 99403809; 2 Department of Clinical Medicine, Aalborg University Hospital, Aalborg, North Denmark, Denmark; 3 Centre for Pancreatic Diseases and Mech-Sense, Department of Gastroenterology and Hepatology, Aalborg University Hospital, Aalborg, Denmark

**Keywords:** compression, continuous glucose monitoring, CGM, encoding, reconstruction, signal, diabetes, data

## Abstract

**Background:**

Continuous glucose monitoring (CGM) generates high-frequency time-series data, creating challenges for efficient storage, transmission, and analysis.

**Objective:**

This study aimed to develop and evaluate a CGM-specific compression method that achieves high compression ratios while preserving signal fidelity and clinically relevant glycemic metrics.

**Methods:**

We introduce a content-based encoding approach (PN+) that represents CGM profiles using physiologically salient landmarks: glucose peaks and nadirs and a small set of optimally selected support points. Reconstruction is performed using piecewise cubic Hermite interpolation. PN+ was evaluated against peaks and nadirs only, uniform downsampling, piecewise aggregate approximation, and autoencoder-based compression. Performance was assessed across multiple compression ratios using 2 complementary datasets: 40,000 synthetic CGM profiles and real-world CGM data from a randomized crossover trial (558 days from 30 patients). Performance was evaluated using compression ratio, mean absolute error, and *R*^2^ between original and reconstructed CGM-derived clinical metrics.

**Results:**

At a compression ratio of 13 (22 points per 24-hour profile), PN+ achieved substantially lower reconstruction error than comparator methods (mean absolute error=0.77 vs 2.75-3.45) and consistently higher *R*^2^ values across glycemic metrics. Improvements were most pronounced for excursion-sensitive measures such as mean amplitude of glycemic excursions, where PN+ reduced error by more than 4-fold compared with downsampling, piecewise aggregate approximation, and autoencoders. These performance advantages were preserved in heterogeneous real-world data. Encoding and decoding required less than 0.2 seconds per profile, supporting practical scalability.

**Conclusions:**

PN+ enables robust CGM data compression by explicitly preserving physiologically meaningful glucose dynamics. The method outperforms generic compression techniques in reconstructing clinically relevant metrics while maintaining low computational overhead, making it well suited for large-scale CGM storage, interoperability, and downstream analytics.

## Introduction

### Background

Continuous glucose monitoring (CGM) has become a cornerstone of modern diabetes care, yielding a time series of glucose measurements every few minutes [1-3]. This high-resolution data stream has revolutionized both clinical management and research, enabling advanced evaluation of therapies, classification of patient subgroups, data-driven prediction of glucose dynamics, and discovery of glycemic patterns via machine learning [4-9]. For example, the adoption of CGM in type 1 diabetes has surged in recent years, reflecting its importance for decision support and personalized treatment [10]. However, the richness of CGM data and other biomedical data also poses challenges [11]: large numbers of daily readings per patient produce very large datasets that must be stored, shared, and processed efficiently.

Storing and interoperating on such high-frequency data can be prohibitive. As Jacobsson et al [11] state, modern health care systems face an “ever-increasing need for retrieving, storing, and managing the large amount of biomedical signal data generated,” and common data exchange standards such as the Health Level Seven Fast Healthcare Interoperability Resources are inefficient when used as a long-term storage format. Naively saving CGM streams as text-based Fast Healthcare Interoperability Resources records or CSV files incurs high overhead and wastes storage. These inefficiencies hinder scalable CGM data integration and slow down analysis. Together, these challenges highlight the need to explore compression or compact encoding methods that reduce data size while preserving critical clinical information [11].

Efficient encoding and compression are also key to secure data sharing and interoperability. In emerging health care architectures, technologies such as blockchain and the Internet of Things require lean data formats to function at scale [12,13]. For instance, blockchain-based platforms for diabetes care have been proposed to improve data sharing and patient-centric control [14], but the limited throughput of distributed ledgers means that raw CGM time series cannot be stored on-chain without preprocessing. By compressing and encoding glucose traces into concise, standardized representations, data can be embedded in interoperable payloads or off-chain repositories and exchanged securely between hospitals and patients.

At the same time, CGM analysis increasingly relies on artificial intelligence and deep learning. Neural networks for glucose forecasting and pattern recognition often require well-structured, fixed-dimensional input and benefit from reduced noise. Textual encoding of CGM traces has been shown to greatly aid such analytics. For example, Igbe et al [15] demonstrated that mapping CGM profiles to reduced-alphabet “strings” could enable powerful search and classification, as well as support predictive modeling, anomaly detection, and even generative artificial intelligence applications. In clinical practice, alphabetic encoding could facilitate data indexing, reduce search time for glycemic patterns, and reduce computational complexity by leveraging text-based data structures and machine learning pipelines. Such encoded biomedical data can be fed directly into deep learning models to improve the speed and accuracy of analysis [16,17]. Several time-series data representations have been proposed for analysis, such as perceptually important points, which reduce dimensionality by dynamically retaining only the critical data points, such as extreme peaks or rapid fluctuations, that govern the primary trajectory of a sequence [18]. Another technique is piecewise aggregate approximation (PAA), which discretizes a raw time series into equal-length segments and replaces each with its arithmetic mean [19], whereas symbolic aggregate approximation further extends this by mapping those aggregated numerical values into a discrete alphabet [20]. Beyond these time-domain heuristics, frequency-domain transformations have proven effective for electrocardiogram (ECG) signals [21]. In addition, methods such as the use of convolutional autoencoders have been applied to ECG signals. This approach leverages the feature extraction power of deep learning to achieve significant data reduction while using a residual mechanism to specifically address and correct the reconstruction errors [22].

### Problem Statement

Despite the rapid growth in the use of CGM, compression algorithms specifically designed for CGM data remain relatively underexplored. Most existing approaches rely on generic signal compression techniques that do not explicitly account for the clinical characteristics of glucose dynamics. CGM exhibits less temporal redundancy in contrast to other biological signals such as ECGs, which makes compression challenging. In this study, we aimed to propose a compression approach that extends the concept of perceptually important points by focusing on peak-nadir structures and strategic support points within CGM signals. The underlying hypothesis was that CGM data can be compressed at high compression ratios while still enabling accurate reconstruction of the signal, preserving clinically relevant information and maintaining low error in commonly used CGM-derived clinical metrics.

### Contributions

This work makes 3 primary contributions. First, we introduce a CGM-specific, content-based compression method (PN+) that encodes glucose profiles using physiologically salient landmarks—peaks, nadirs, and a small set of optimally selected support points—enabling high compression while preserving clinically meaningful signal dynamics. Second, we provide a comprehensive quantitative evaluation of PN+ across multiple compression ratios by comparing it with uniform downsampling, PAA, and autoencoder-based compression using both synthetic and real-world CGM datasets and demonstrate better fidelity for excursion-sensitive consensus CGM metrics. Third, we show that high-fidelity reconstruction of CGM signals is achievable from a sparse landmark representation with minimal computational overhead.

## Methods

### Overview

**Figure 1.**
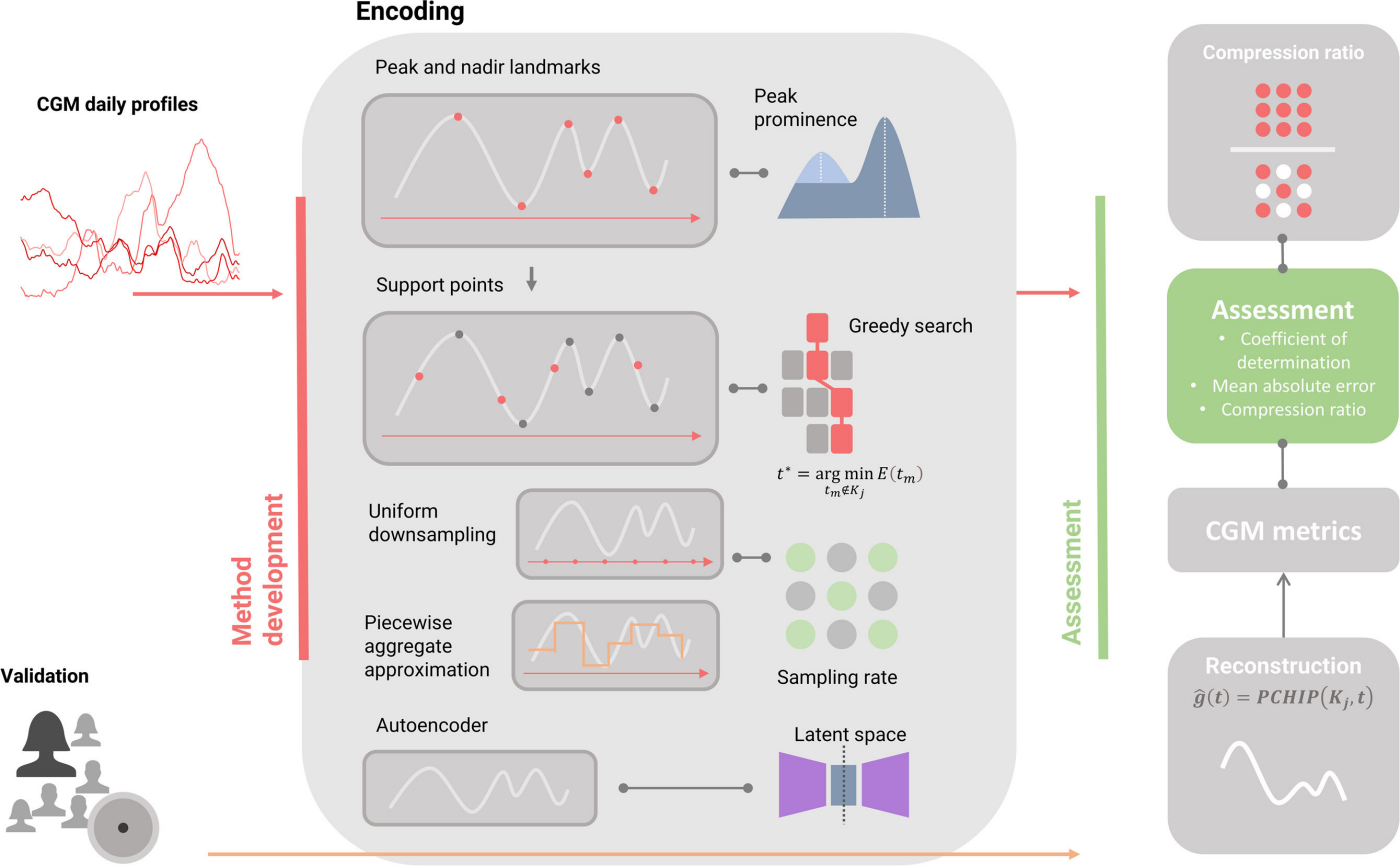
Overview of the methodological framework. Continuous glucose monitoring (CGM) daily profiles were encoded using four approaches: (1) peak and nadir landmarks identified by peak prominence, (2) support points selected through a greedy search procedure, (3) uniform downsampling at a predefined sampling rate, and (4) autoencoder-based compression. The encoded signals were subsequently reconstructed using piecewise cubic Hermite interpolating polynomial (PCHIP). Reconstruction performance was evaluated based on the coefficient of determination, mean absolute error, and compression ratio.

We used 2 complementary CGM datasets—synthetic data generated via a conditional generative adversarial network (CGAN) [23,24] and real-world CGM measurements from a randomized crossover trial [25]—to develop and validate 2 encoding methods based on peak and nadir information and on peaks and nadirs with support points and compare them with uniform downsampling, autoencoder, and PAA approaches. The encoding reduced each profile to a small set of values, and reconstruction of the CGM signal used interpolation. We compared compression ratios, reconstruction error (mean absolute error; MAE), and established CGM glycemic metrics between original and decoded signals. Statistical analyses assessed whether encoding preserved clinically relevant glucose features. An overview of the methodology is illustrated in Figure 1.

### Ethical Considerations

All procedures were performed in compliance with relevant laws and institutional guidelines and have been approved by the appropriate institutional committee. For the validation data, the North Denmark Region Committee on Health Research Ethics approved the protocol (N-20210064), and the study adhered to the Declaration of Helsinki and good clinical practice. Written informed consent was obtained before enrollment.

### Data Sources

#### Discovery Data

We used a publicly available synthetic CGM dataset [23,24] generated by a CGAN trained on real CGM time series from healthy individuals and patients with type 1 diabetes across 4 hemoglobin A_₁c_ groups (<6.5%, 6.5% to <7%, 7% to <8%, and ≥8%). In brief, the CGAN architecture was based on a conditional on-class label (hemoglobin A_₁c_ group), with generator and discriminator networks following the formulation by Mirza and Osindero [26]. The dataset included 40,000 profiles of 24 hours each with a sample frequency of 288 measurements per day.

#### Clinical Validation Data

For validation, we used CGM baseline recordings from a randomized, open-label, crossover trial in patients with chronic pancreatitis and insulin-treated diabetes (N=30; mean age 64.4, SD 8.8 years; 75.9% male), each undergoing 20 days of CGM (Dexcom G6; sampling rate: 5-minute intervals) for the baseline [25]. The dataset consisted of 558 days, each treated as an independent observation in the analysis. The patient cohort was characterized by high inter- and intraglycemic variability, making it a relevant population for evaluating compression methods in CGM data with low temporal redundancy [27,28].

### Encoding and Decoding Algorithms

We compared 5 encoding approaches for each CGM profile. For each signal landmark (peak, nadir, or support point), we recorded its time stamp and glucose value.

#### Peaks and Nadirs Only

This method involved identifying local maxima (“peaks”) and minima (“nadirs”) by sign changes in the discrete derivative of the glucose time series subject to a minimum prominence threshold to avoid noise.

#### Peaks, Nadirs, and Support Points (PN+)

This method extended the peak and nadir approach by incorporating additional “support” points between peaks and nadirs. These points were selected using a greedy search strategy to better capture the overall shape of the glucose trajectory.

#### Uniform Downsampling

As a baseline comparison, we applied uniform downsampling by removing every *x*th sample to simulate a reduced sampling rate.

#### Autoencoders

A deep neural network autoencoder architecture [29] was implemented as a comparison approach using an encoder-decoder framework. The network was trained for 800 epochs with L2 and sparsity regularization. Five-fold cross-validation was used to minimize overfitting. The latent space dimensionality was chosen to achieve compression ratios comparable to those of the other methods.

#### PAA Method

The CGM time series was partitioned into a predefined number of contiguous, nonoverlapping segments of approximately equal length. Within each segment, glucose values were replaced with their mean, yielding a compressed representation that preserved the overall temporal trend while reducing high-frequency variability. The number of segments was selected to achieve a compression ratio comparable to those of the other methods, and reconstruction was performed by assigning each segment mean back to its corresponding time interval.

### Signal Reconstruction

Decoded profiles were reconstructed using piecewise cubic Hermite interpolating polynomial (PCHIP) [30] using the identified landmarks, yielding a time series equivalent to the original 5-minute sampling rate. This simple method preserves key excursion patterns while minimizing computational load.

### Peak Detection in Glucose Time Series

To identify the significant excursions in glucose levels, we used a peak detection algorithm. Glucose values were treated as a 1D time series *g*(*t*), where *t* represents the corresponding time stamps of glucose measurements.

Local maxima were identified with a minimum peak prominence threshold of 15 mg/dL to ensure physiological relevance and reduce the influence of minor fluctuations and noise. In this context, prominence quantifies how much a peak stands out due to its height and separation from neighboring valleys. Specifically, the prominence of each peak was computed as the vertical distance between the peak and the lowest point in the signal, separating it from a higher neighboring peak.

The detection criterion can be formally described as that each candidate peak at time *t_i_* was retained only if the following applied:


$$g\left(\;t_{i}\right)-max\left(min_{t_{k}<t_{i}}g_{k}^{↓},min_{t_{k}>t_{i}}g_{k}^{↓}\right)\geq 15\;mg/dL$$

In this equation, $g_{k}^{↓}$ represents local valleys in the signal. This approach yielded a set of clinically relevant glucose peaks. For nadir detections, the same procedure was repeated with an inverted signal.

### Including Additional Support Points

To efficiently approximate CGM profiles using the limited number of representative landmarks, we implemented a greedy optimization strategy for selecting support points. The goal was to identify a sparse subset of additional points that allowed for the accurate reconstruction of the original signal using PCHIP.

Given an initial subset of *k* known landmark points *K*_0_={(*t_i_*_1_, *g_i_*_1_), ..., (*t_ik_*, *g_ik_*)} where *i_j_* ∈ {1, ..., *N*}, the goal was to iteratively augment this set to a total of *n* points by selecting additional time points that minimized reconstruction error. The upper bound for *n* was constrained by the desired compression ratio.

At each iteration *j*, the algorithm evaluated all remaining candidate time points *t_m_* ∉ *K_j_* and selected the one that, when added, minimized the L2 norm of the reconstruction error:


$$E_{t}(t_{m})={\left\|g(t)-\hat{g}(K_{j},t_{m})\right\|}_{2}$$

In this equation, *g*(*K_j_*; *t_m_*) is the interpolated glucose signal using PCHIP over the updated key set *K_j_*_+1_ ∪ {(*t_m_*, *g*(*t_m_*))}. The optimal new point was selected as follows:


$$t^{*}=\underset{t_{m}\notin K_{j}}{argmin}E\left(t_{m}\right)$$

This process was repeated until |*K_j_*| reached the maximum number of desired landmark points. The final reconstructed signal *g*(*t*) was generated via *g*(*t*)=PCHIP (*K_j_*, *t*).

**Figure 2.**
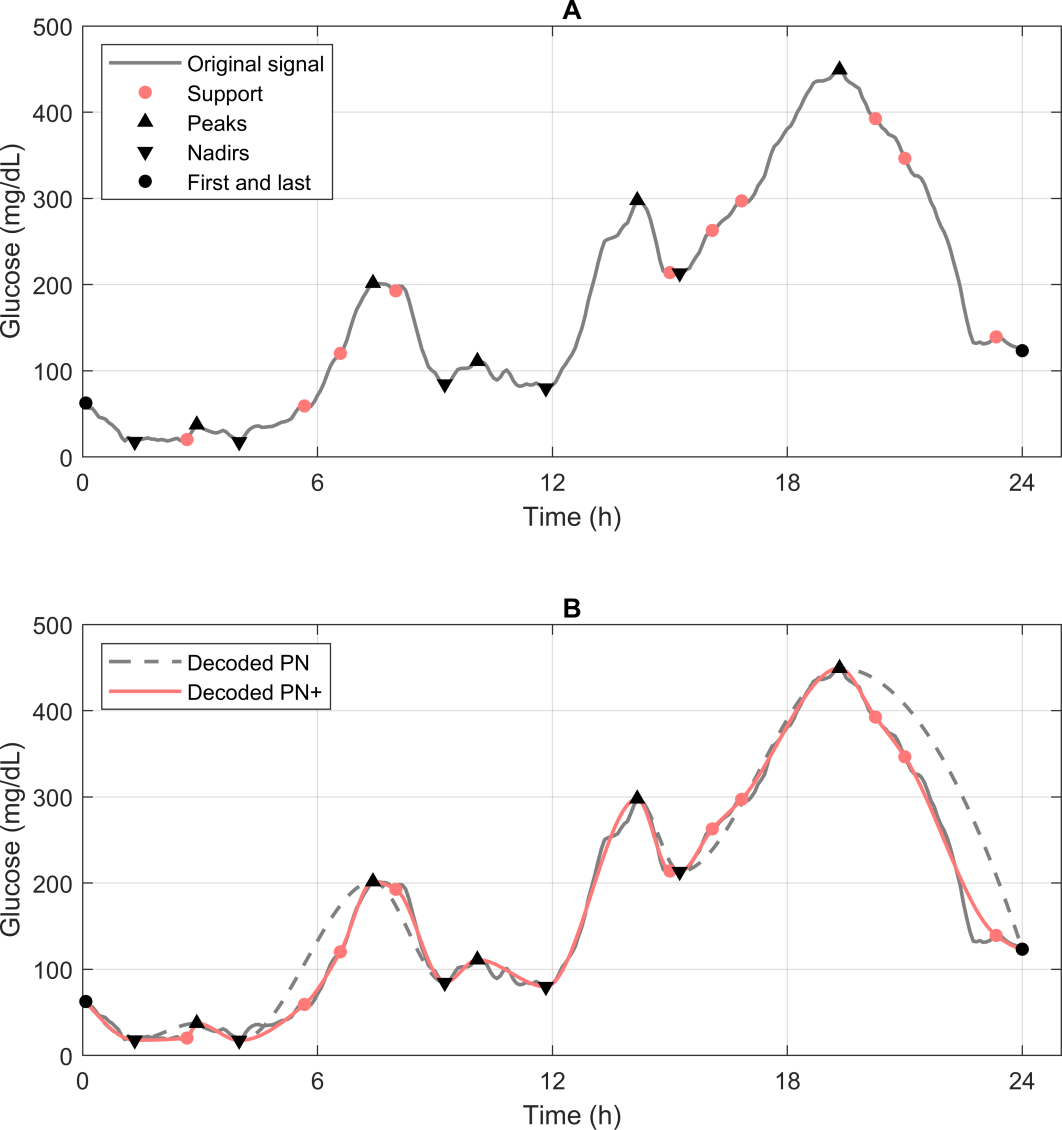
(A) An example of a 24-hour continuous glucose monitoring profile (original signal) with the peak, nadir, and support points identified by the algorithm and (B) the reconstructed signals using only the peak and nadir points (PN) and using the method with additional support points (PN+).

Figure 2 illustrates the selected landmark points extracted from the CGM profiles, including both peak and nadir points as well as the additional support points used in the extended PN+ approach. The corresponding reconstructed signals for both methods are shown. Notably, the inclusion of support points in the PN+ approach improved the alignment of the reconstructed signal with the original glucose profile, particularly in regions between peaks and nadirs where the rate of change was nonuniform.

### Assessments

To assess the compression of the approaches (peaks and nadirs only, PN+, downsampling, autoencoders, and PAA), we calculated the compression ratio between the uncompressed and compressed profiles:


$$Compression\;ratio=\;\frac{uncompressed}{compressed}$$

Furthermore, we calculated the MAE between the original and decoded glucose profile metrics:


$$MAE=\;\frac{1}{N}\sum_{i=1}^{n}|metric{\left(\;P_{i}\right)}_{orginal}-metric{\left(\;P_{i}\right)}_{reconstructed}|$$

To assess whether the encoding profiles preserved clinically relevant glucose features, we computed standard consensus CGM metrics and glycemic variability metrics [31-33] on both original and decoded profiles. The MAE and coefficient of determination (*R*^2^) were calculated for each metric between the original and decoded profiles. The standard consensus CGM metrics included were mean glucose, SD of glucose, coefficient of variation, time in range (70-180 mg/dL), time in tight range (70-140 mg/dL), time below range (TBR; <70 mg/dL), TBR level 1 (54-70 mg/dL), TBR level 2 (<54 mg/dL), time above range (TAR; >180 mg/dL), TAR level 1 (180-250 mg/dL), TAR level 2 (>250 mg/dL), and mean amplitude of glycemic excursions (MAGE) [34].

Analyses were performed in MATLAB (vR2021b; MathWorks Inc) on a laptop equipped with an 11th-generation Intel Core i7-11850H (2.50 GHz), 32 GB of RAM, and an NVIDIA T1200 graphics processing unit. Standard toolboxes for signal processing and deep learning were used, and all analyses were performed on this configuration to ensure the reproducibility of the results. For computing CGM metrics, we used an open-source tool, Quantification of Continuous Glucose Monitoring, designed for CGM data analysis using the MATLAB environment [35].

## Results

### Discovery Data

**Figure 3.**
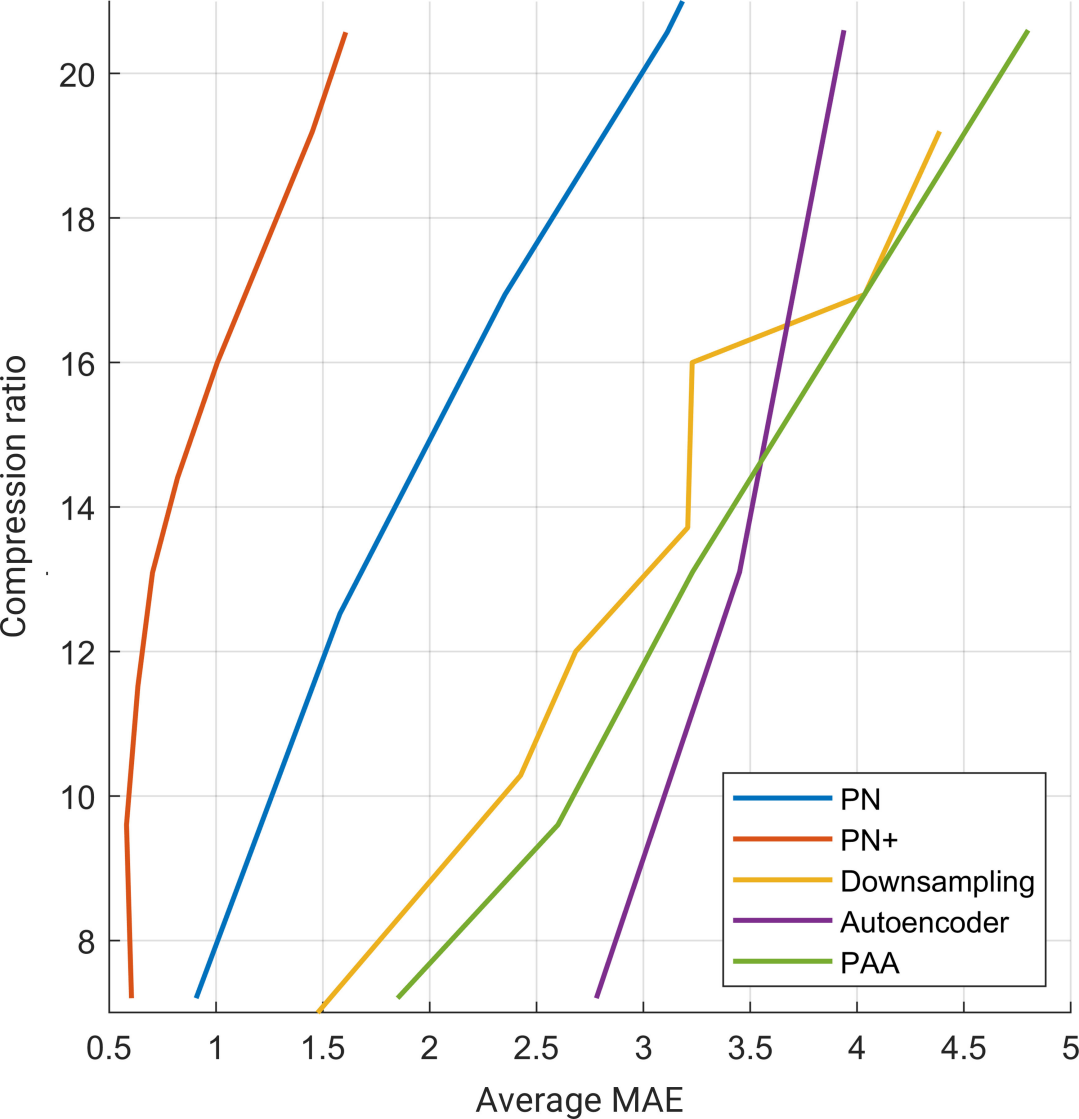
Compression ratio plotted against the average mean absolute error (MAE) values for the 4 methods across all glycemic metrics derived from the reconstructed continuous glucose monitoring signals. PAA: piecewise aggregate approximation; PN: peaks and nadirs only; PN+: peaks and nadirs and support points.

**Figure 4.**
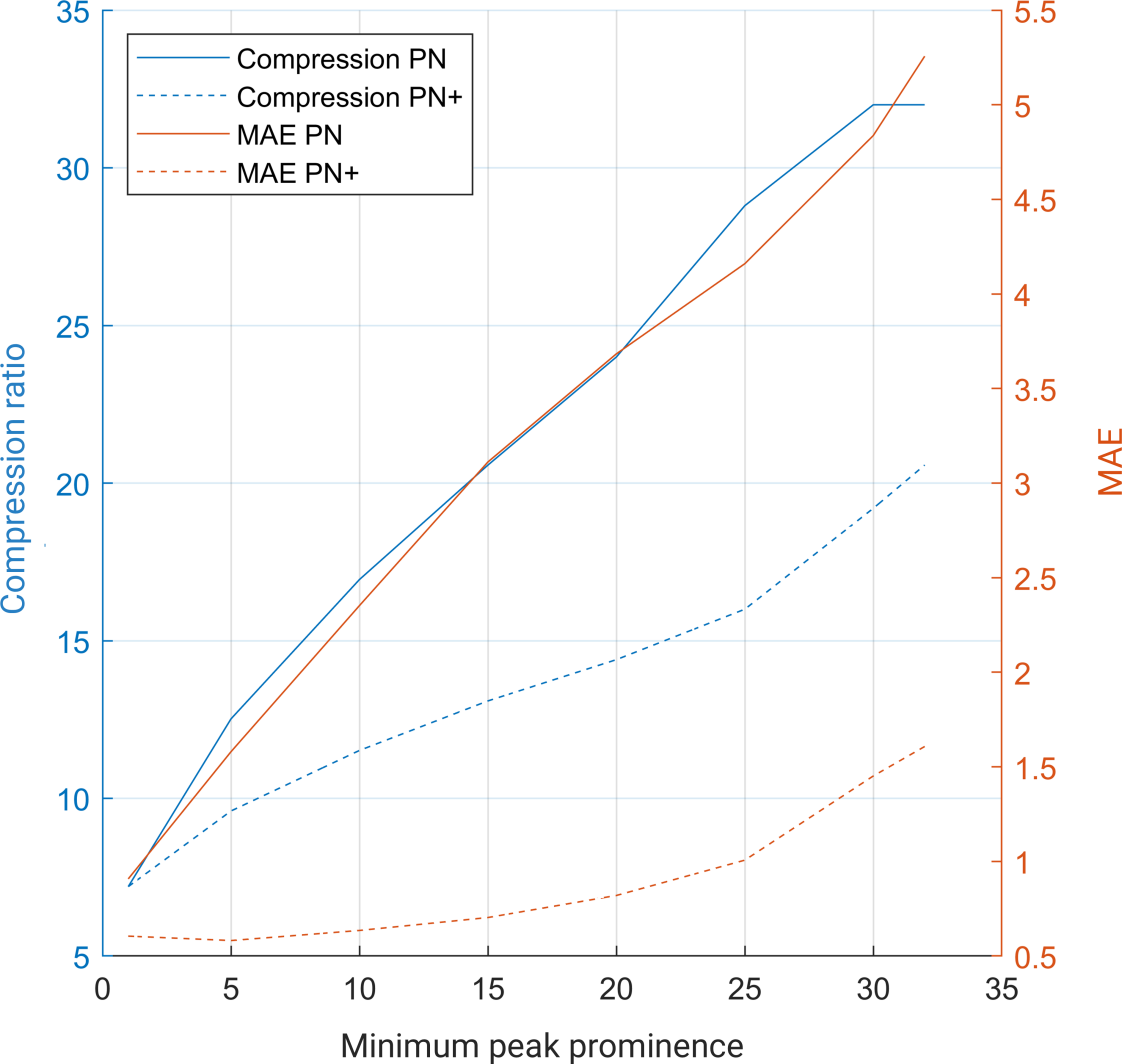
Impact of varying peak prominence thresholds on the mean absolute error (MAE) of the peaks and nadirs only (PN) and peaks and nadirs with support points (PN+) approaches.

As shown in Figure 3, the methods were compared across various settings—downsampling rate, latent space dimensions, peak prominence threshold, and the allowed number of total support points—to evaluate performance under different compression rates. Among the methods, PN+ consistently achieved the highest average *R*^2^, whereas downsampling yielded the lowest performance at each comparable compression ratio. Furthermore, the impact of varying peak prominence thresholds on the peaks and nadirs only and PN+ approaches is illustrated in Figure 4. As expected, PN+ resulted in lower compression at each threshold level due to the inclusion of additional support points. However, when comparing the methods at equivalent compression ratios, PN+ exhibited substantially lower error than the peaks and nadirs only method. This suggests that PN+ is a more effective approach for capturing clinically relevant physiological signal patterns. The encoding and decoding combined took an average of 0.13 (SD 0.05) seconds per 24-hour profile on a PC laptop (11th-generation Intel Core i7-11850H at 2.50 GHz, 32 GB of RAM).

**Figure 5.**
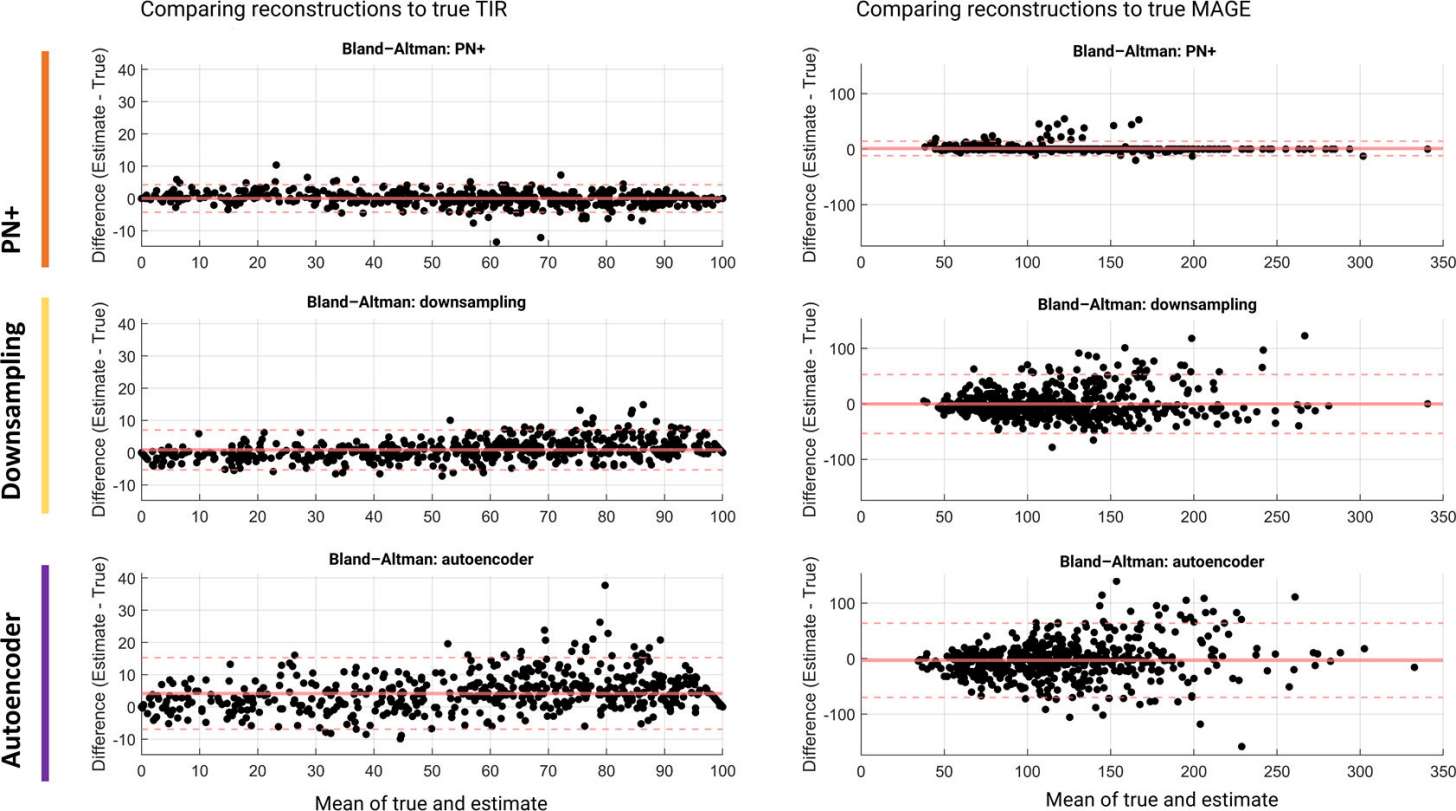
Bland-Altman analysis plots comparing the methods (peaks, nadirs, and support points [PN+], downsampling, and autoencoders) for reconstruction error estimates of clinical metrics (time in range [TIR] and mean amplitude of glycemic excursions [MAGE]) on clinical validation data.

A direct comparison of CGM-derived metrics from the reconstructed signals and the original signals is shown in Table 1 using a peak prominence threshold of 15 mg/dL and a minimum compression ratio of 13 (corresponding to 22/288, 7.6% of the original landmark points). At this compression level, PN+ consistently achieved higher *R*^2^ values across most CGM-derived metrics than the downsampling, autoencoder, and PAA methods. More notably, the MAE for each metric was substantially lower with PN+, with a reduction factor of 3.6 to 4.5 (Table 1). This improvement was especially pronounced for MAGE, which captures large glucose excursions—an area where the downsampling and autoencoder methods introduced significant estimation errors, as illustrated in Figure 5 through a Bland-Altman analysis plot.

Overall, PN+ demonstrated strong alignment between metrics derived from reconstructed and original signals. However, the estimation of TBR level 2, representing rare low-glucose episodes, remained sensitive to the encoding and decoding process due to the sparse occurrence of such events over a typical day.

**Table 1.** Overall performance and compression ratio of peaks and nadirs only (PN) encoding, PN encoding with support points (PN+), downsampling, autoencoders, and piecewise aggregate approximation (PAA) for each continuous glucose monitoring metric in the discovery dataset (n=40,000).

	PN^a^		PN+^b^		Downsampling^b^		Autoencoder^b^		PAA^b^	
	*R* ^2^	MAE^c^	*R* ^2^	MAE	*R* ^2^	MAE	*R* ^2^	MAE	*R* ^2^	MAE
Mean glucose (mg/dL)	0.98	5.24	1	0.64	1	0.61	1	1.98	1	0.14
CV^d^ (ratio)	0.97	2.01	1	0.50	0.99	2.02	0.97	2.08	1	1.42
SD of glucose (mg/dL)	0.98	3.3	1	0.65	1	2.91	0.99	2.84	1	1.99
TIR^e^ (%)	0.97	3.36	1	1.14	0.99	2.12	0.97	3.83	0.99	2.43
TITR^f^ (%)	0.97	3.64	1	1.36	0.99	2.36	0.98	4.45	0.99	2.68
TBR^g^ (%)	0.90	1.52	0.98	0.66	0.97	1.1	0.87	2.5	0.98	1.15
TBR1^h^ (%)	0.75	1.36	0.92	0.82	0.89	1.11	0.64	1.95	0.92	1.32
TBR2^i^ (%)	0.90	0.66	0.98	0.32	0.96	0.47	0.75	1.42	0.98	0.5
TAR^j^ (%)	0.97	2.79	1	0.70	0.99	1.38	0.99	1.63	0.99	1.83
TAR1^k^ (%)	0.92	2.76	0.99	0.89	0.97	1.87	0.96	2.14	0.98	2.34
TAR2^l^ (%)	0.96	1.89	1	0.40	0.99	0.98	0.99	1.03	0.99	1.25
MAGE^m^ (mg/dL)	0.97	3.71	0.99	1.1	0.90	16.13	0.89	15.55	0.92	21.76
Overall mean	0.94	2.69	0.99	0.77	0.98	2.75	0.92	3.45	0.96	3.23

a Compression ratio: 20.6.

b Compression ratio: 13.1.

c MAE: mean absolute error.

d CV: cross-validation.

e TIR: time in range.

f TITR: time in tight range.

g TBR: time below range.

h TBR1: TBR level 1.

i TBR2: TBR level 2.

j TAR: time above range.

k TAR1: TAR level 1.

l TAR2: TAR level 2.

m MAGE: mean amplitude of glycemic excursions.

### Clinical Validation Data

As shown in Table 2, the validation dataset yielded overall performance trends similar to those observed in the discovery dataset. However, the *R*^2^ and MAE values were slightly worse across all methods, likely reflecting the increased complexity and variability inherent in real-world patient data. Notably, the validation cohort included individuals with diabetes associated with chronic pancreatitis, a diabetes subtype known for markedly impaired glucose regulation and high glycemic variability due to deficiencies in insulin and glucagon secretion along with variable insulin sensitivity [27]. Despite this added complexity, PN+ continued to outperform the downsampling, autoencoder, and PAA methods at equivalent compression ratios, achieving the lowest error and highest *R*^2^ among the compared methods.

**Table 2.** Overall performance and compression ratio of peaks and nadirs only (PN) encoding, PN encoding with support points (PN+), downsampling, autoencoders, and piecewise aggregate approximation (PAA) for each continuous glucose monitoring metric in the clinical validation dataset.

	PN^a^		PN+^b^		Downsampling^b^		Autoencoder^b^		PAA^b^	
	*R* ^2^	MAE^c^	*R* ^2^	MAE	*R* ^2^	MAE	*R* ^2^	MAE	*R* ^2^	MAE
Mean glucose (mg/dL)	0.96	8.13	1	0.99	1	1.4	0.99	5.78	1	0.17
CV^d^ (ratio)	0.87	2.68	0.99	0.69	0.97	1.68	0.93	2.24	1	1.25
SD of glucose (mg/dL)	0.89	5.16	0.99	1.15	0.98	2.9	0.95	4.38	1	2.15
TIR^e^ (%)	0.93	6.26	0.99	1.43	0.99	2.32	0.98	5.16	1	2.4
TITR^f^ (%)	0.89	5.84	0.99	1.63	0.98	2.34	0.97	5.34	0.99	2.3
TBR^g^ (%)	0.75	0.95	0.95	0.33	0.90	0.49	0.8	1.02	0.96	0.45
TBR1^h^ (%)	0.60	0.80	0.92	0.31	0.91	0.39	0.79	0.81	0.94	0.47
TBR2^i^ (%)	0.75	0.41	0.84	0.16	0.56	0.16	0.16	0.21	0.82	0.14
TAR^j^ (%)	0.94	5.65	1	1.24	0.99	2.01	0.99	4.24	1	2.2
TAR1^k^ (%)	0.79	5.83	0.98	1.57	0.96	2.89	0.92	5.45	0.98	2.88
TAR2^l^ (%)	0.95	3.56	1	0.81	0.99	1.5	0.99	2.6	1	1.56
MAGE^m^ (mg/dL)	0.90	5.79	0.98	1.86	0.72	20.2	0.79	24.32	0.81	23.41
Overall mean	0.85	4.25	0.97	1.01	0.91	3.19	0.78	5.12	0.92	3.28

a Compression ratio: 24.1.

b Compression ratio: 13.1.

c MAE: mean absolute error.

d CV: cross-validation.

e TIR: time in range.

f TITR: time in tight range.

g TBR: time below range.

h TBR1: TBR level 1.

i TBR2: TBR level 2.

j TAR: time above range.

k TAR1: TAR level 1.

l TAR2: TAR level 2.

m MAGE: mean amplitude of glycemic excursions.

## Discussion

### Principal Findings

The proposed novel encoding algorithm PN+ demonstrated advantages for CGM data compression by explicitly selecting physiologically meaningful points. By anchoring the compressed signal on glucose peaks and nadirs (and additional support points via a greedy strategy), PN+ yielded substantially lower reconstruction error and higher explained variance (*R*^2^) across CGM-derived metrics than the uniform downsampling, PAA, and autoencoder methods at equivalent compression ratios. The method consistently preserved clinical metrics across both the discovery and clinical validation datasets. Performance improvements were observed across multiple glycemic measures, and the methods demonstrated resilience to variations in signal patterns, including postprandial excursions. In particular, PN+ better preserved MAGE, a gold-standard CGM metric for assessing large glycemic excursions [36]. Because MAGE and similar variability indexes depend on accurately capturing excursions, PN+’s targeted point selection translates directly to better metric fidelity. Furthermore, reconstructing the compressed signal using PCHIP maintains the original shape and smoothness of glucose trends [35], avoiding the overshoot or oscillation that might arise with simpler interpolation. Overall, the proposed algorithm was able to reconstruct the CGM signals, preserving key clinically relevant features of the curves from a substantially decompressed version. While the deep learning–based comparison method, the autoencoder, enables nonlinear encoding and decoding of the CGM signal and represents a more advanced data-driven compression strategy, it did not translate into improved signal reconstruction with respect to clinically derived metrics in our experiment. The implemented autoencoders were constrained by the latent space dimensionality to achieve compression ratios comparable to those of the other evaluated methods. The downsampling approach performed comparably in preserving clinical metrics that were not sensitive to variability; however, it was less effective in capturing dynamic metrics compared with PN+.

To date, relatively few studies have addressed the compression or encoding of CGM profiles. However, Kovatchev et al [37], using a multistep machine learning procedure, showed how it is possible to reconstruct a virtual CGM profile from the original sparse data (7-point blood glucose profiles) while preserving clinically relevant information [38]. Notably, Igbe and Kovatchev [15] recently introduced a novel approach that encodes daily CGM profiles into symbolic representations—referred to as CGM strings and CGM texts—that preserve key clinical metrics while achieving data compression. Their analysis demonstrated that a 9-character encoding corresponding to an approximate compression ratio of 10:1 could retain information on cross-validation with an *R*^2^ of up to 0.93. The method presented in this study builds on and extends these findings by enabling the preservation of a broader range of CGM-derived metrics across multiple compression levels. Moreover, our approach offers the additional advantage of reconstructing the original signal with high precision, supporting both clinical interpretability and downstream analytical use.

The proposed approach is fundamentally a content-based compression strategy and could be applied to other periodic or episodic biomedical signals. For example, ECG waveforms hinge on distinct QRS complex peaks for heartbeats, and compression methods that preserve those peaks can maintain diagnostic integrity. Indeed, prior work has shown that a lossy ECG compression scheme preserved the main features of the ECG morphology (notably, the QRS complexes) despite a high compression ratio of 4.5 [39]. Similarly, photoplethysmographic waveforms depend on capturing the systolic pulse peaks; a PN+–like method could retain these pulse peaks as key support points. In both ECG and photoplethysmography contexts, the idea is the same as for CGM: allocate sampling “budget” to physiologically salient events (heartbeats or pulses) and interpolate the rest. Thus, PN+ is broadly relevant to any biosignal where preserving the height and timing of key peaks and nadirs is critical for clinical or functional interpretation. Future work should explore the potential applicability of the proposed methodology to other biological signals. Moreover, future work should also investigate the clinical impact on decision-making based on reconstructed signal data.

### Strengths and Limitations

This study was based on a large synthetic dataset of CGM profiles derived from a heterogeneous population of individuals with diabetes. Furthermore, the proposed methods were externally validated using data from a distinct patient group with diabetes secondary to chronic pancreatitis. This could support the generalizability of the results to a broader population of individuals with diabetes. However, the proposed PN+ method has some limitations typical of lossy, feature-based compression approaches. One concern is rare event sensitivity: very short or abrupt excursions (such as sudden severe hypoglycemia events) might not always produce easily identified peaks or may occur between selected support points. In practice, we observed that extremely brief dips can be slightly underrepresented by PN+ unless thresholds are tuned to catch them. Relatedly, the performance gain of PN+ depends on the presence of signal structure. If a CGM trace is unusually flat or noisy (eg, due to sensor artifacts), the greedy peak-nadir selection may yield fewer benefits, and overall reconstruction error for all methods can increase. Indeed, in our heterogeneous validation cohort, all compression methods saw modestly reduced accuracy, although PN+ remained superior; this suggests that real-world variability poses challenges even to structured approaches. Finally, PN+ currently uses fixed rules (greedy selection and threshold-based peak and nadir identification), so it may require adaptation (eg, dynamic thresholds or rule changes) when applied to data with very different characteristics.

### Conclusions

The proposed PN+ method produces a compact CGM representation that retains critical glycemic dynamics while discarding redundant portions of the signal. The CGM signal can be reconstructed with high precision from the encoding representation. The application of the method to other biomedical signals needs further investigation.

## Abbreviations

CGAN: conditional generative adversarial network
CGM: continuous glucose monitoring
ECG: electrocardiogram
MAE: mean absolute error
MAGE: mean amplitude of glycemic excursions
PAA: piecewise aggregate approximation
PCHIP: piecewise cubic Hermite interpolating polynomial
TAR: time above range
TBR: time below range
